# Avoiding prepositional pile-up

**DOI:** 10.1007/s40037-015-0200-1

**Published:** 2015-07-30

**Authors:** Lorelei Lingard

**Affiliations:** Schulich School of Medicine & Dentistry, Health Sciences Addition, Western University, Rm. 112, N6A 5C1 London, ON Canada

In the writer’s craft section we offer simple tips to improve your writing in one of three areas: Energy, Clarity and Persuasiveness. Each entry focuses on a key writing feature or strategy, illustrates how it commonly goes wrong, teaches the grammatical underpinnings necessary to understand it and offers suggestions to wield it effectively. We encourage readers to share comments on or suggestions for this section on Twitter, using the hashtag: #how’syourwriting?

What’s wrong with the following sentence?

After receiving research ethics approval from the review boards at three teaching hospitals affiliated with two universities in urban Ontario, Canada, we asked clinical and educational leaders in each hospital to identify inter-professional clinical health care teams with reputations for strong collaborations and managerial/administrative support.

This sentence is long, but there’s no golden rule about sentence length. It’s complex, but it doesn’t commit any grammatical errors. Its main clause is well-formed, with a clear subject (*we*) and an active main verb (*asked*). It’s properly punctuated. Then why is it so hard to digest?

Diagnosis: prepositional pile-up. This sentence—my own! [1]—features nine prepositions:

**After** receiving research ethics approval **from** the review boards **at** three teaching hospitals affiliated **with** two universities **in** urban Ontario, Canada, *we asked clinical and educational leaders* **in** each hospital **to** identify inter-professional clinical health care teams **with** reputations **for** strong collaborations and managerial/administrative support.

These prepositions require the reader to sift through a host of details that elaborate the main clause—*we asked clinical and educational leaders*.

What’s a preposition? The most common prepositions are the ‘little words’: *in, to, with, on, at, of, for*. Prepositions show relationships of: direction (e.g., *from, to*), place (e.g., *under, over, in*), time (e.g., *before, after, during, until*), cause (e.g., *by, of, in order that*), manner (e.g., *with, among, against*) and amount (e.g., *most of, both of*) [2]. Prepositions sit at the beginning of ‘prepositional phrases’ which include the preposition’s object: *on* *the test*; *from* *the review board*; with *two universities*; *in* *urban Ontario, Canada*.

Prepositional phrases serve an important function, allowing the writer to provide additional detail about the main action. But a writer must ask herself, are these additional details really necessary? And if they are necessary, is a pile of prepositional phrases the best way to present them? In my sentence, five prepositional phrases provide additional detail about the main action before the reader encounters that main action:

**After** *receiving research ethics approval*—Ethics approval from where?—**from** *the review boards*—Which review boards?—**at** *three teaching hospitals*—Which hospitals?—*affiliated* **with** *two universities*—Where were these universities?—**in** *urban Ontario, Canada*, *we asked clinical and educational leaders* …

Such chains of prepositional phrases zoom in closer and closer on detail. But because they accumulate before the main clause, they can distract the reader from the central thrust of the sentence.

As Pinker explains, ‘The human mind can only do a few things at a time, and the order in which information comes in affects how that information is handled’ (p. 83) [2]. According to this logic, prepositional pile-up early in a sentence risks overwhelming the reader. It can do so, for instance, by creating a long noun phrase in the subject position (underlined), which directly precedes the main verb. The following is an example:

*The attempt* ***to*** *articulate milestones relevant* ***to*** *both specific activities* ***of*** *practice and stages* ***of*** *trainee development* *is* **at** the centre **of** our current competency-based assessment activities.

Long noun phrases in the subject position are often followed by a form of the verb *to be (is)*. As discussed in a previous Writer’s Craft section, the verb *to be* can drain energy from your prose [3]. The following revision addresses both the prepositional pile-up and the weak verb:

Competency-based assessment requires the articulation **of** milestones reflecting both specific practice activities and training stages.

The revision reduces the number of prepositional phrases, partly by converting phrases like *stages* **of** t*rainee development* to *training stages*. But it also moves the main subject and verb*—Competency-based assessment requires—*to earlier in the sentence, so that the reader is anchored by the main action before encountering layers of detail.

Fixing prepositional pile-up is not simply a matter of purging prepositions from your writing. Prepositions are necessary, and we rarely craft a sentence without them. (In this article, only four sentences are preposition-free.) When revising, you need to identify prepositions that can be removed without changing meaning (*stage* **of** *trainee development → training stage*) and ascertain whether preposition placement is taxing the reader’s attention. Sometimes, the best solution to prepositional pile-up is to break the sentence into two. My first example requires this revision technique:

We received research ethics approval **from** three urban teaching hospitals **in** Ontario, Canada. *Clinical and educational leaders* were asked **to** identify inter-professional health care teams reputed **to** be collaborative and administratively well-supported.

Why do prepositions pile up in academic writing? Perhaps because, in order to meet journal word length requirements, writers try to cram as much detail into as few words as possible. (It’s no accident that my first example comes from a methods section; I was probably trying to detail the nuances of the study sample as efficiently as I could.) Preposition-heavy writing may give writers a false sense that they are using words efficiently, when in fact they are both adding to the word count and creating verbal quicksand for the reader. Now that you know what prepositional pile-up looks like and how it affects readers, you can lighten the prepositional load in your writing.
